# AAV9-Mediated Overexpression of TRPM4 Increases the Incidence of Stress-Induced Ventricular Arrhythmias in Mice

**DOI:** 10.3389/fphys.2019.00802

**Published:** 2019-06-27

**Authors:** Andy Pironet, Ninda Syam, Frone Vandewiele, Chris Van den Haute, Sara Kerselaers, Silvia Pinto, Greetje Vande Velde, Rik Gijsbers, Rudi Vennekens

**Affiliations:** ^1^Laboratory of Ion Channel Research, TRP Research Platform Leuven, VIB Center for Brain and Disease, Department of Cellular and Molecular Medicine, KU Leuven, Leuven, Belgium; ^2^Molecular Virology and Gene Therapy, Department of Pharmaceutical and Pharmacological Sciences, KU Leuven, Leuven, Belgium; ^3^Biomedical MRI, Department of Imaging & Pathology, KU Leuven, Leuven, Belgium

**Keywords:** ion channels, TRPM4, cardiac arrhythmias, conduction disorders, triggered activity, telemetry, AAV9

## Abstract

Ca^2+^ activated non-selective (CAN) cation channels have been described in cardiomyocytes since the advent of the patch clamp technique. It has been hypothesized that this type of ion channel contributes to the triggering of cardiac arrhythmias. TRPM4 is to date the only molecular candidate for a CAN cation channel in cardiomyocytes. Its significance for arrhythmogenesis in living animals remains, however, unclear. In this study, we have tested whether increased expression of wild-type (WT) TRPM4 augments the risk of arrhythmias in living mice. Overexpression of WT TRPM4 was achieved via tail vein injection of adeno-associated viral vector serotype 9 (AAV9) particles, which have been described to be relatively cardiac specific in mice. Subsequently, we performed ECG-measurements in freely moving mice to determine their *in vivo* cardiac phenotype. Though cardiac muscle was transduced with TRPM4 viral particles, the majority of viral particles accumulated in the liver. We did not observe any difference in arrhythmic incidents during baseline conditions. Instead, WT mice that overexpress TRPM4 were more vulnerable to develop premature ventricular ectopic beats during exercise-induced β-adrenergic stress. Conduction abnormalities were rare and not more frequent in transduced mice compare to WT mice. Taken together, we provide evidence that overexpression of TRPM4 increases the susceptibility of living mice to stress-induced arrhythmias.

## Introduction

The Transient Receptor Potential Melastatin member 4 (TRPM4) is a Ca^2+^ activated non-selective (CAN) cation channel (Launay et al., 2002; Nilius et al., 2003). The role of CAN channels in the heart muscle is somewhat illustrious. Considering that these channels are active when the intracellular Ca^2+^ concentration is increased, they might contribute to action potential (AP) duration and Na^+^ influx during the AP. Furthermore, considering that spontaneous Ca^2+^ release events are an important trigger mechanism for cardiac arrhythmias, it might be anticipated that CAN cation channels contribute to after depolarizations, or determine cellular excitability in this context (Cannell and Lederer, 1986; Ehara et al., 1988; Giles and Shimoni, 1989).

We and others have previously shown that TRPM4 is expressed and functionally active in cardiomyocytes (Demion et al., 2007; Abriel et al., 2012; Kruse and Pongs, 2014; Mathar et al., 2014). The cardiac expression level of TRPM4 is the highest in Purkinje fibers followed by atrial and ventricular cardiac myocytes (Fonfria et al., 2006; Kruse et al., 2009; Mathar et al., 2010, 2014). TRPM4 is activated during AP repolarization and loss of the Trpm4 gene (*Trpm4*^–/–^) results in a shortened ventricular AP (Mathar et al., 2014). Cardiac features of Trpm4^–/–^ mice include increased ß-adrenergic reserve and increased hypertrophic growth of the cardiac muscle after chronic Angiotensin II treatment and endurance training (Mathar et al., 2014; Kecskes et al., 2015; Gueffier et al., 2017). Jacobs et al. (2015) studied the disease pattern of wild-type (WT) and Trpm4^–/–^ mice after obstruction of the left anterior descending artery, resulting in severe ischemia of the cardiac muscle and heart failure. Here, Trpm4^–/–^ mice showed improved survival, and the Trpm4^–/–^ cardiac muscle showed increased contractility after ß-adrenergic stimulation.

Finally, numerous mutations in the human Trpm4 gene were associated with hereditable conduction disorders (for reviews, see Abriel et al., 2012; Watanabe et al., 2013; Baruteau et al., 2015; Guinamard et al., 2015). Analysis of the first described mutation, E7K, suggested a gain-of-function due to increased SUMOylation of the mutant channel protein, leading to impaired endocytosis and an increase in the number of functional channels in the plasma-membrane (Kruse et al., 2009). Instead, others described gain-of-function of TRPM4 resulting from aberrant Ca^2+^-dependent deactivation in the A432T mutant channel (Xian et al., 2018).

However, to date, *in vivo* data on the consequences of TRPM4 gain-of-function are lacking. Following up on the concept of Kruse et al. (2009), we tested whether overexpression of TRPM4 *per se* predisposes living mice to heart rhythm abnormalities. To this end, we used tail-vein injection of adeno-associated viral vector serotype 9 (AAV9) particles encoding TRMP4. AAV vectors are widely used as transgene expression delivery means (Zincarelli et al., 2008; Van der Perren et al., 2011; Choi et al., 2014). Its use is preferred over adenoviruses, herpesviruses, and lentiviruses due to its low immunogenicity and persistent expression (Wright et al., 2001; Vandendriessche et al., 2007; Zincarelli et al., 2008). Among all available AAV-serotypes, AAV9 has the best cardiotropic properties (Wright et al., 2001; Vandendriessche et al., 2007; Zincarelli et al., 2008).

## Materials and Methods

### Animals

Male WT and *Trpm4*^–/–^ mice were used for all experiments. All mice were housed in a conventional animal facility with regular white/dark cycle with 12 h of light and unrestricted access to food and water. All experiments were approved by the local ethical committee for animal experiments.

### DNA Construct

We constructed two TRPM4-containing AAV transfer plasmids with and without Woodchuck Hepatitis Virus Posttranscriptional Regulatory Element (WPRE). Full-length mouse TRPM4 cDNA (3639 bp) was amplified from our plasmid collection with XbaI and SpeI sites inserted at 5′ and 3′ ends, respectively. Primer sequences used for amplification are as followed: XbaI_TRPM4_Mm_F 5′-AAA TCT AGA ATG GTG GGG CCG GAG AAG GA-3′, SpeI_TRPM4_Mm_R 5′-TTT ACT AGT CTA TTA TCA GTC TTT GGA ACC AGT GG-3′. After appropriate digestion, XbaI-mTRPM4-SpeI amplicon was then subcloned into pAAV-TF-CMV-intron-GFP-MCS substituting its GFP portion while retaining WPRE. In the second construct, WPRE was removed by MluI and BglII before the same procedure was applied. Both constructs were subsequently verified by test digest and sequencing.

### AAV9-TRPM4 Production

AAV viral vectors were produced as described in Van der Perren et al. (2011) with minor modifications. Briefly, HEK 293T cells were transfected using 25-kDa linear polyethylenimine (PEI) with the AAV transfer plasmids, AAV2/9 rep/cap and pAdDeltaF6 in a ratio of 1:1:1. HEK cells were seeded in a Hyperflask Cell Culture Vessel (1720 cm^2^) at 1.3 × 10^8^ cells per production in DMEM supplemented with 2% fetal calf serum. The next day 400 μg of each plasmid was transfected by generation of a DNA-PEI complex for 15 min and adding to the medium. After 24 h of incubation at 37°C the medium was replaced by Optimem without fetal calf serum. The medium was collected 5 days after transient transfection and concentrated using tangential flow filtration. The vector present in the concentrated medium was purified using a discontinuous iodixanol step gradient of 20, 30, 40, and 60%. The gradient was centrifuged at 27 000 (or 27,000 see below) rpm for 2 h in a Beckman Ti-70 fixed angle rotor. Gradient fractions were collected in 250 μl aliquots and fractions with a refraction index between 1.39 and 1.42 were pooled and further concentrated in a Vivaspin 6 filter. The iodixanol was exchanged with PBS and vector aliquots were stored at −80°C.

Viral vector titers were determined using real-time PCR with a primer probe set for the polyA sequence and displayed as genome copies/mL (GC/mL). The three relevant vector preparations and their yields were: AAV2/9_CMV-intron-TRPM4(-WPRE) 2.12 × 10^11^ GC/ml, AAV2/9_CMV-intron-TRPM4(+WPRE) 1.32 × 10^11^ GC/ml and AAV2/9_CMV-intron-eGFP-T2A-fLuc-WPRE 5.20 × 10^10^ GC/ml. These are further referred to as AAV2/9_CMV-intron-TRPM4 and AAV2/9_CMV-intron-TRPM4-WPRE and AAV2/9_CMV-intron-eGFP-T2A-fLuc-WPRE, respectively.

### Transduction

Wild-type and *Trpm4*^–/–^ male mice aged 16–18 weeks old were anesthetized with ketalar (75 mg/kg body weight; Pfizer, Brussels, Belgium) and domitor (1 mg/kg body weight; Pfizer, Brussels, Belgium) and their tails were dipped in warm water for better exposure of the vein prior to injection. WT mice were randomized to receive 1.7 × 10^9^ genome copies of either AAV2/9_CMV-intron-TRPM4(-WPRE) or AAV2/9_CMV-intron-eGFP-T2A-fLuc-WPRE via tail vein injection using 30G syringe needle (Becton Dickinson, Franklin Lakes, NJ, United States) Mice were laid on a warm bed during the procedure. Their anesthesia was reversed with antisedan (0.5 mg/kg body weight; Orion Pharma, Newbury, Berkshire, United Kingdom). Before use in experiments, mice were in quarantine for 2 weeks according to biosafety regulations.

### Telemetric ECG-Measurements

To measure ECGs in awake, freely moving animals, ECG transmitters (PhysioTel model TA11ETA-F10, DataSci, New Brighton, MN, United States) were implanted in 10–12 weeks old male WT or Trpm4^–/–^ mice (Background = C57Bl6/N). Briefly, the animals were anesthetized with isoflurane (induction: 5% inhaled isoflurane in 1000 cmł/min O_2_, maintenance: 1.5–2% inhaled isoflurane in 500 cm^3^/min O_2_) and body temperature was maintained at 37°C by a heating pad (Harvard Apparatus, Holliston, MA, United States). The transmitter was inserted in the intraperitoneal (ip) cavity and ECG leads were fixed subcutaneously on the thorax (i.e., 1 cm to the left of the sternum) and on the right pectoral muscle, to achieve a Lead II configuration. Mice received analgesics [0.05 mg/kg buprenorphine (ip), Ecophar] after surgery and were monitored daily during a 1-week recovery. All telemetric recordings were performed in a closed black box to minimize the effect of the environment on the behavior of the animal with a 12-h day/night cycle and data was recorded with the Ponemah Physiology Platform v.5.0 (DataSci, New Brighton, MN, United States). Telemetric ECGs were recorded at a sampling frequency of 2.5 kHz.

To evoke cardiac arrhythmias, mice were stressed by exercise test combined with an intraperitoneal administration of 1 mg/kg isoprenaline. On day 1–3, mice underwent a habituation and two consecutive training sessions on a horizontal Exer-6M treadmill (Colombus Instruments, Columbus, OH, United States). On day 4, an exercise test was performed and mice had to run until exhaustion, which was defined when mice failed six times to jump back on the treadmill. In this case, the speed of the treadmill was decreased, allowing them to get back on the treadmill again. After first failure, the speed of the treadmill was not increased anymore. Complete exhaustion was defined when the speed had to be adapted for the sixth time. After complete exhaustion, mice were extra stimulated via 1 mg/kg isoprenaline, placed in the black box and ECG was measured and analyzed for 1 h.

### ECG Analysis

ECG measurements in the Lead II configuration were analyzed offline by Ponemah Physiology Platform v5.0 (DataSci, New Brighton, MN, United States) and Labchart 7 (ADInstruments, New Zealand). In baseline conditions, the ECG was analyzed for ECG-parameters and incident of cardiac arrhythmias for 1 h during the 12-h night cycle. The overall heart rate was determined on 10 s intervals every 10 min. To determine ECG-parameters in nocturnal baseline conditions, a 30 s block of ECG-traces without electrical noise or motion artifacts, and with a HR around 550 bpm was analyzed. The following parameters were determined: P-wave duration, PR-interval, QRS-interval, and QT-interval (Figure 4A). The analysis was performed via the ECG-analysis module of Labchart7. It averages 10 consecutive beats and positions of the marks were manually checked before final calculations of the intervals. The QT-interval was corrected using the Mitchell formula (Mitchell et al., 1998).

Analysis of cardiac arrhythmias was manually performed. Briefly, the heart rate was checked for sudden deflections. Subsequently, it was determined whether these result from arrhythmic complexes or a misinterpretation/miscalculation of the positioning of the QRS-mark by the software due to motion/lead artifacts. Motion/lead artifacts were distinguished from ECG-traces by frequency and morphology of the signal. These artifacts occur typically at much higher frequency then the average R–R interval and are recognized as narrow electrical spikes fluctuating from the iso-electric line. These artifacts were excluded from the dataset. Incidents of ventricular arrhythmias and conduction disturbances were analyzed for 1 h in nocturnal baseline conditions (2–3 AM) and for 1 h after the exercise stress test. Conductions disturbances were grouped since electrical noise often made it impossible to distinguish different events. Typical examples were 2nd-degree atrio-ventricular (AV) blocks, AV-blocks with escape beats and sinus pauses (Figure 5). Sinus pauses were defined when PP-interval was longer than twice the baseline sinus cycle. Definitions for the determinations of ventricular arrhythmias were based on the Lambeth Conventions II (Curtis et al., 2013). Ventricular arrhythmias were divided in single ventricular ectopic beats (VEBs), couplet-, triplet-VEBs, non-sustained ventricular tachycardia (VT; run of 4 to 10 consecutive VEBs), sustained VT (run of 10 or more consecutive VEBs), idioventricular tachycardia, and ventricular fibrillation (Figure 5).

### Membrane Protein Isolation and Western Blot

One whole mouse heart was homogenized in 250 μl of 1x Lysis buffer (50 mM HEPES; 150 mM NaCl; 1.5 mM MgCl2; 1 mM EGTA pH8; 10% Glycerol; 1x Protease Inhibitor cocktail without EDTA Roche, Basel, Switzerland; pH = 7.4) with Polytron manual disperser (Kinematica, Luzern, Switzerland) on ice. One volume of the same lysis buffer containing 2% Triton X-100 was added to the homogenate and mixed. Subsequently, five volumes of Saccharose buffer (250 mM Saccharose; 10 mM HEPES; 1x Protease Inhibitor cocktail without EDTA; pH = 7.4) was added to the homogenate and lysed on a rotating wheel for 2 h at 4°C. The lysate was then centrifuged at 3000 g for 15 min at 4°C, and the resulting supernatant was transferred to a new tube for further ultracentrifugation at 200,000 *g* for 40 min at 4°C. The pellet containing membrane proteins was then resuspended in 200 μl of Saccharose buffer and quantified with BCA protein assay (Thermo Fisher, Waltham, MA, United States). Eighty micrograms of each sample was loaded on 4–15% TGX gel (Biorad, Hercules, CA, United States) and blotted onto PVDF membrane (Biorad, Hercules, CA, United States). Anti-TRPM4 AK578 1:500 (a gift from Prof. Veit Flockerzi, Homburg, Germany) and anti-Na^+^/K^+^ ATPase α1 ab7671 1:5 000 (Abcam, Cambridge, United Kingdom) antibodies were used for the immunoblot. Band density for TRPM4 and Na^+^/K^+^-ATPase was determined using ImageJ (Schneider et al., 2012).

### Bioluminescence Imaging

Mice were imaged in an IVIS Spectrum *in vivo* Imaging System (PerkinElmer, Waltham, MA, United States). Before imaging, the mice were anesthetized in an induction chamber using isoflurane (induction: 2% in 1000 cm^3^/min O_2_; during imaging: 1.5% in 500 cm^3^/min O_2_). Prior to *in vivo* imaging, 126 mg/kg D-luciferin (Promega, Leiden, Netherlands) dissolved in PBS (15 mg/ml) was intraperitoneally injected into the mice. Afterward, they were positioned in the IVIS and consecutive images were acquired until the maximum signal (photon flux through a region of interest covering the organ of interest) was reached. Immediately after *in vivo* imaging, these mice were sacrificed by cervical dislocation and harvested for their heart, liver, kidney, and lungs. These organs were then imaged *ex vivo* with the same system.

### Statistics

All statistical analyses were performed using Origin9 (Microcal Origin, United States) and Prism (Graphpad, United States). Data is shown as mean ± SEM. Normality was checked via a Shapiro–Wilk test and data was considered normally distributed if *p* > 0.05. Differences between two groups were detected using the non-parametric Mann–Whitney test. Differences between three groups were detected using a 1-Way ANOVA or its non-parametric alternative Kruskall–Wallis ANOVA followed by a Dunn’s post-test. Differences in paired groups were detected using a pair-sample *t*-test or its non-parametric alternative Pair Sample Wilcoxon Signed Rank Test. A *p*-value < 0.05 was considered as statistically significant.

## Results

### AAV9-Based Viral Vector Can Accommodate Up to ∼5.1 kb to Deliver a Functional Protein

**FIGURE 1:**
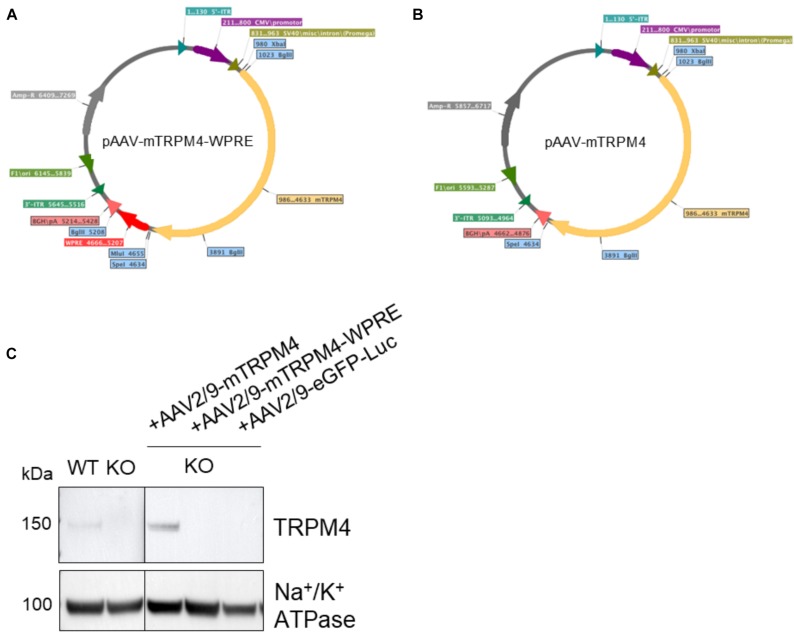
Maps of plasmid constructs generated for this study. **(A)** cDNA of mouse TRPM4 was subcloned into XbaI and SpeI restriction sites resulting in a 5645 bp fragment between 5′-ITR and 3′-ITR. **(B)** After removal of WPRE, cDNA of mouse TRPM4 was subcloned into the same XbaI and SpeI restriction sites resulting in a shorter fragment of 5093 bp between 5′-ITR and 3′-ITR. **(C)** The two constructs were produced as viral particles and injected via tail vein into TRPM4^–/–^ mice to asses their functional expression. AAV2:9-eGFP-Luciferase was also included here. Each sample loaded (80 ug) was from membrane fraction isolation.

The efficiency of AAV particle production and eventually the functionality of protein expression that it delivers, depends on the total size including 5′-ITR and 3′-ITR together with the presence of enhancing elements, such as the WPRE and polyadenylating signal (Loeb et al., 1999; Choi et al., 2014). Therefore, its packaging size can be a limitation for its usefulness, and it was suggested that for AAV2 any vectors exceeding 4.9 kb showed reduced expression (Dong et al., 1996). Since mouse TRPM4 cDNA alone is quite large (3639 bp), we employed two strategies for its subcloning into the AAV transfer plasmid. In one plasmid, WPRE was retained giving a total size of 5645 bp (Figure 1A), while in the other transfer construct, the WPRE was removed resulting in a smaller size of 5093 bp (Figure 1B). Both plasmid constructs were transfected into HEK293T cells to verify protein expression and were confirmed to produce functional TRPM4 channels by patch clamp (not shown).

Subsequently, with these two constructs we tested whether either the size limit or the WPRE enhancer element is more important. Both versions of AAV9 vector particles were subsequently produced, referred to as AAV2/9_CMV-TRPM4 and AAV2/9_CMV-TRPM4-WPRE, respectively (AAV2: vector backbone; AAV9: viral vector capsid serotype). Vector production efficiency was not significantly affected with GC/ml being in the same order of magnitude for both rAAV vector preps (2.12 × 10^11^ GC/ml and 1.32 × 10^11^ GC/ml for AAV2/9_CMV-TRPM4 and AAV2/9_CMV-TRPM4-WPRE, respectively).

To verify functionality of the viral vector constructs, the resulting vectors were next injected (1.7 × 10^9^ GC) via the tail vein into *Trpm4*^–/–^ mice and as a control, AAV2/9_CMV-eGFP-T2A-fLuc-WPRE was injected. As is clear from Figure 1C, AAV9 without WPRE was able to induce expression of TRPM4 in the heart of *Trpm4*^–/–^ mice. While the construct without WPRE even showed higher expression of TRPM4 compared to WT, the one with WPRE failed to do so. We concluded that for optimal expression, genomic size limitation is more important than the presence of the enhancer element. In our design, AAV9 vector can accommodate up to a total size of 5.1 kb.

### AAV2/9_CMV-TRPM4 Delivers TRPM4 Into the Mouse Liver and Heart

**FIGURE 2:**
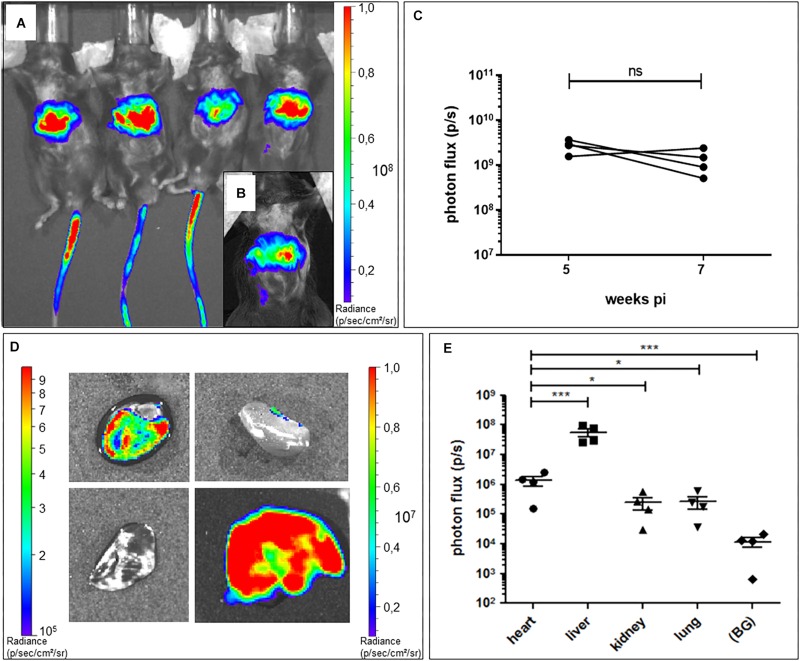
Bioluminescence imaging of AAV2:9-mediated Luciferase-expression in heart and liver of WT mice. **(A)** *In vivo* bioluminescence imaging at 5 weeks and **(B)** 7 weeks post tail vein injection (pi) of AAV2/9_CMV-eGFP-T2A-fLuc-WPRE in WT mice. **(C)** *In vivo* ventral photon flux of the same mice at 5 and 7 weeks pi of AAV2/9_CMV-eGFP-T2A-fLuc-WPRE (WT+Luc: *n* = 4; Mann–Whitney test on log-transformed data) **(D)** *Ex vivo* bioluminescence imaging of isolated organs [heart (h) with apex pointing toward left bottom corner, kidney (k), lungs (lu), and liver (li)] of a representative mouse at 7 weeks pi. Note the different luminescence scale for the liver (right scale) compared to the other organs (left scale). **(E)** *Ex vivo* bioluminescence signal intensity of isolated organs at 7 weeks pi (WT+Luc: *n* = 4; repeated measures ANOVA followed by a Dunn’s test on the log-transformed data, ^*^p < 0.05, ^∗∗∗^*p* < 0.001).

Different serotypes of AAV virus are known to target different organs in animals, and AAV9 has been reported to be relatively specific for the heart in mouse, depending on the number of virus particles applied and the route of administration (Wright et al., 2001; Zincarelli et al., 2008). Based on these previous studies, we chose AAV2/9 tail-vein injection to deliver murine Trpm4 cDNA for expression in the mouse heart. To test in which organs it delivers the gene of interest, we used AAV2/9_CMV-eGFP-T2A-fLuc-WPRE. Firefly luciferase reporter activity following D-luciferin injection can be conveniently and non-invasively monitored over time with a bioluminescence imager in living animals. Mice were injected with 1.7 × 10^9^ GC via the tail-vein. 5 weeks after injection, whole animal imaging in living animals was applied to quantify the luciferase-induced photon flux. As shown in Figure 2A, a strong signal is obvious in the tail (from the position where the tail-vein injection was performed and upward), the upper abdominal area and none in the upper left thorax (Figure 2A). 2 weeks later (7 weeks post-injection), a strong signal was observed in the upper abdominal area and none in the upper left thorax (Figure 2B). Ventral photon flux was the same at 5 weeks pi and 7 weeks pi (Figure 2C). Subsequently, we harvested liver, lungs, kidney, and heart for *ex vivo* imaging and compared the photon flux for the respective organs (Figure 2D). Obviously, the high luciferase signal in the upper abdominal area originated from the liver (Figure 2E). In the kidney, luminescence was significant. In the lungs, luminescence was virtually absent. In the heart, luminescence was obvious in all chambers, though atria consistently were less luminiscent then ventricles. This likely results from the lower number of cells and cell layers in the atrial fraction compared to the ventricular fraction. Taken together, these data indicate that AAV2/9_CMV-eGFP-T2A-fLuc-WPRE particles can indeed transduce the cardiac muscle, but is by no means “cardiac-selective.”

### TRPM4 Protein Expression in the Heart From AAV2/9_CMV-TRPM4 Injected Mice

**FIGURE 3:**
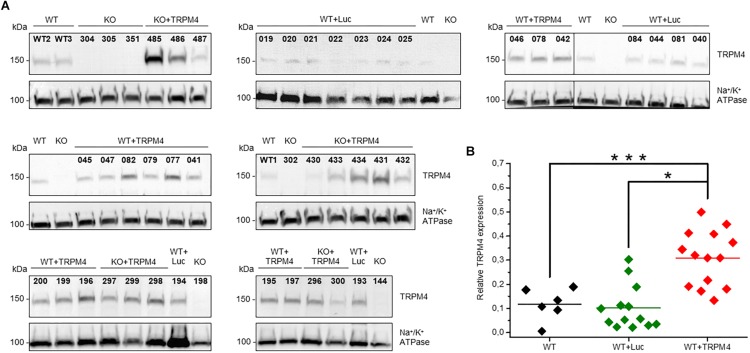
Protein expression level of TRPM4 transduced by AAV9 from WT and TRPM4^–/–^ (KO) mouse hearts. **(A)** Each mouse whole heart was used for membrane fraction isolation, and 80 ug was loaded into SDS-PAGE gel. Immunoblots were revealed with anti-TRPM4 and anti Na^+^/K^+^ ATPase α1 antibodies. **(B)** Relative expression of TRPM4 compared to Na^+^/K^+^-ATPase (WT: *n* = 6, WT+Luc: *n* = 13, WT+TRPM4: *n* = 14; Kruskall-Wallis ANOVA followed by a Dunn’s post test, ^*^p < 0.05, ^∗∗∗^*p* > 0.001).

We evaluated the level of TRPM4-expression delivered by AAV2/9_CMV-TRPM4 viral particles by Western blotting. We injected AAV2/9_CMV-TRPM4 by tail-vein into WT and *Trpm4*^–/–^ mice, and harvested whole heart for membrane protein isolation. Figure 3A summarizes all western blots of animals that were used in this study (numbers above each lane indicate the personal identifier for each injected animal). Analysis indicates that TRPM4 protein expression was successful in mouse hearts from both WT and *Trpm4*^–/–^ mice. Though varying levels of TRPM4 expression are obvious, they were consistently higher in hearts of animal injected with AAV2/9_CMV-TRPM4 (both in WT and *Trpm4*^–/–^ mice) than the respective uninjected WT and *Trpm4*^–/–^ hearts and WT hearts subjected to AAV2/9_CMV-eGFP-T2A-fLuc-WPRE (Figure 3B). Mean TRPM4 expression level (expressed as ratio of TRPM4:Na,K-ATPase) in uninjected WT was 0.11808 ± 0.02717 (*n* = 5), in AAV2/9_CMV-TRPM4 (WT+TRPM4) 0.3087 ± 0.03057 (*n* = 14) and in AAV2/9_CMV-eGFP-T2A-fLuc-WPRE (WT+Luc): 0.10252 ± 0.2555 (*n* = 13).

### Mice Overexpressing TRPM4 Are More Prone to Develop Ventricular Arrhythmias Under β-Adrenergic Stress

**FIGURE 4:**
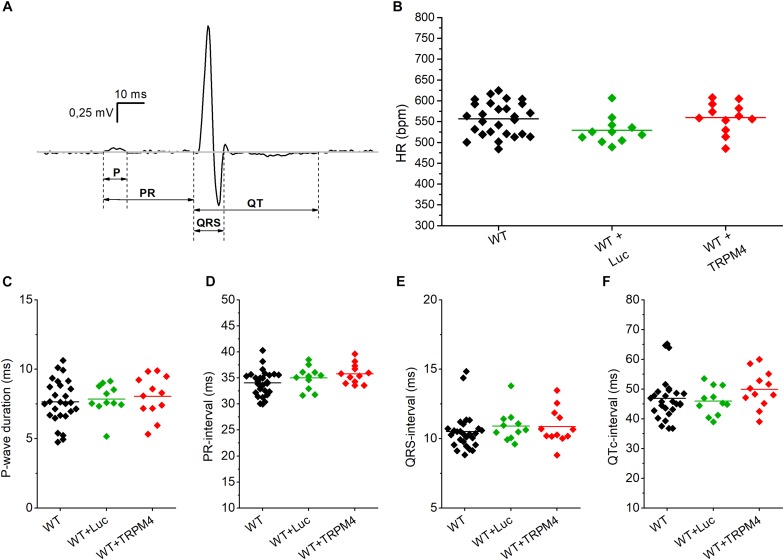
Conduction parameters in freely moving WT, WT+Luc, and WT+TRPM4 mice (WT: *n* = 27, WT+Luc: *n* = 11, WT+TRPM4: *n* = 12). **(A)** Representative ECG-trace with indicated conduction intervals. **(B–F)** Comparison of heart rate **(B)**, P-wave duration **(C)**, PR-interval **(D)**, QRS-interval **(E),** and corrected QT-interval **(F)** between awake WT, WT+Luc, and WT+TRPM4 mice (One-way ANOVA: HR and p-wave duration; Kruskall–Wallis ANOVA followed by a Dunn’s post test: PR-, QRS-, QTc-interval).

We measured the cardiac phenotype of WT mice injected with AAV2/9_CMV-TRPM4 (1.7 × 10^9^ GC) *in vivo* via telemetric ECG. Non-injected WT mice and AAV2/9_CMV-eGFP-T2A-fLuc-WPRE injected mice were used as controls. We found no differences in ECG parameters between these mouse strains in baseline conditions (Figure 4 and Table 1). Heart rate, P-wave duration, PR-interval, QRS-interval, and QTc-interval were essentially the same for the respective experimental groups.

**TABLE 1:** Conduction parameters from freely moving WT, WT+Luc, WT+TRPM4, TRPM4^–/–^, TRPM4^–/–^+Luc, and TRPM4^–/–^+TRPM4 mice (WT: *n* = 27, WT+Luc: *n* = 11, WT+TRPM4: *n* = 12, TRPM4^–/–^: *n* = 7, TRPM4^–/–^+TRPM4: *n* = 5, One-way ANOVA or Kruskall–Wallis ANOVA).

	**Heart rate**	**P-wave duration**	**PR-interval**	**QRS-interval**	**QT-interval**	**QTc-interval**
	**(bpm)**	**(ms)**	**(ms)**	**(ms)**	**(ms)**	**(ms)**
WT	557 ± 8	7.6 ± 0.4	34.0 ± 0.6	10.8 ± 0.3	50.0 ± 2.2	47.1 ± 1.9
WT+Luc	529 ± 10	7.9 ± 0.3	35.0 ± 0.7	10.9 ± 0.3	48.4 ± 1.3	46.0 ± 1.4
WT+TRPM4	560 ± 11	8.0 ± 0.4	35.8 ± 0.5	10.9 ± 0.4	53.3 ± 2.2	49.9 ± 1.8
TRPM4^–/–^	606 ± 19	8.8 ± 0.8	34.0 ± 1.2	10.6 ± 0.4	55.1 ± 3.4	50.7 ± 2.8
TRPM4^–/–^+TRPM4	605 ± 17	8.9 ± 0.6	34.9 ± 1.5	12.0 ± 1.6	51.4 ± 4.0	48.2 ± 2.8

**FIGURE 5:**
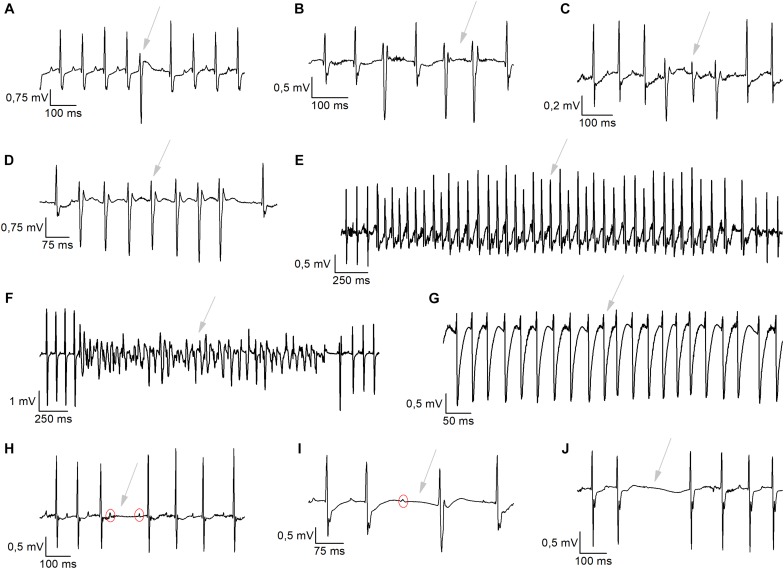
Typical arrhythmic incidents exhibited during nocturnal baseline measurements or during exercise-induced β-adrenergic stress. **(A–G)** Ventricular arrhythmias were detected like single ventricular ectopic (VEB) beats **(A)**, Couplet VEBs **(B)**, Triplet VEBs **(C)**, non-sustained ventricular tachycardia **(D)**, sustained ventricular tachycardia **(E)**, ventricular fibrillation **(F),** and idioventricular rhythm **(G)**. **(H–J)** Representative traces of conductions disturbances, like 2nd-degree atrioventricular blocks **(H)**, AV-block + escape beats **(I),** and sinoatrial arrests **(J)**.

**FIGURE 6:**
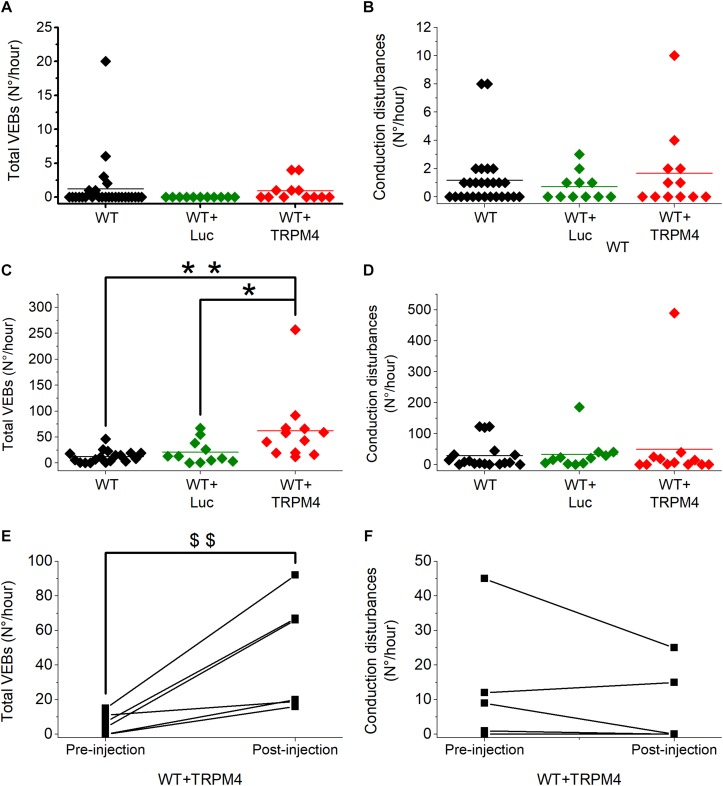
AAV2:9-mediated TRPM4 overexpression increases total number of premature ventricular ectopic beats (VEBs) in freely moving mice during exercise-induced β-adrenergic stress. Solid line indicates mean value. **(A,B)** Total number of VEBs **(A)** and conduction disturbances **(B)** in freely moving WT, WT+Luc, and WT+TRPM4 mice during 1-h nocturnal baseline conditions (WT: *n* = 27, WT+Luc: *n* = 11, WT+TRPM4: *n* = 12; Mann–Whitney test). **(C,D)** Comparison of total number of VEBs **(C)** and conduction disturbances **(D)** in WT, WT+Luc, and WT+TRPM4 mice during exercise-induced β-adrenergic stress (WT: *n* = 18, WT+Luc: *n* = 11, WT+TRPM4: *n* = 12; Kruskall–Wallis followed by a Dunn’s post test, ^*^p < 0.05, ^∗∗^*p* < 0.01). **(E,F)** Paired analysis of total number of VEBs **(E)** and conduction disorders **(F)** before and after injection of TRPM4 during exercise-induced β-adrenergic stress (*n* = 6 for each group; Paired *t*-test, ^$$^*p* < 0.01).

We analyzed 1 h of telemetric measurements in freely moving mice, to detect spontaneous cardiac arrhythmias during the night (Figure 5). All types of arrhythmic events we observed in these mice are illustrated in Figure 5. These events included: conduction disturbances (sino-atrial blocks, 2nd degree atrioventricular (AV) blocks and 2nd degree AV-blocks accompanied with an escape beat); and premature VEBs (single VEBs, couplet-, triplet-VEBs, non-sustained ventricular tachycardia (VT), and sustained VT). As is clear from Figures 6A,B, WT mice rarely developed ventricular arrhythmias or conduction disorders in unstressed, nocturnal baseline conditions. The most common type of tachycardia were single VEBs. One mouse developed one couplet VEB. In addition, the occurrence of either type of arrhythmias was not increased in WT+AAV2/9_CMV-eGFP-T2A-fLuc-WPRE and WT+AAV2/9_CMV-TRPM4 mice in stress-free baseline conditions (Figures 6A,B). The majority of mice in all experimental groups display a low number of events, while some individual mice in each strain are prone to develop more severe arrhythmias.

To provoke cardiac arrhythmias, we subjected mice to an exercise test. To this end, mice were forced to run on a treadmill until exhaustion, upon which they received a bolus of isoprenaline (1 mg/kg). Telemetric ECGs was recorded continuously during the test, until 1 h after isoprenaline injection. Arrhythmias were detected based on Lambeth II conventions, and typical examples are included in Figure 5. As shown in Figures 6C,D, the total number of VEBs, but not conduction disturbances, was significantly larger in WT mice which received AAV2/9_CMV-TRPM4, compared to uninjected WT mice and WT mice that received AAV2/9_CMV-eGFP-T2A-fLuc-WPRE. Compared to unstressed conditions, mice developed more single VEBs and became more prone to complexed tachycardia, like couplet VEBs, triplet VEBs, non-sustained, and sustained VT. Of note, the latter two mouse experimental groups were not significantly different from each other. Interestingly, one animal that received AAV2/9_CMV-TRPM4 developed ventricular fibrillation for 2 s (Figure 5F). This same mouse also developed an idioventricular rhythm for 15 min, corresponding to approximately 9500 beats, after which sinus rhythm recovered (Figure 5E).

To extend these results, we compared the number of arrhythmias associated with the exercise test before and after viral vector transduction in the same mice. As shown in Figure 6E, in mice that received AAV2/9_CMV-TRPM4 the total number of VEBs was significantly higher after the injection, compared to pre-injection. The number of conduction disturbances was not significantly different (Figure 6F).

### Varying TRPM4 Overexpression Levels and Sites May Contribute to the Severity of Arrhythmic Events

As is obvious from Figure 3, the TRPM4 overexpression level of AAV2/9_CMV-TRPM4 injected WT mice was quite variable, as assessed in Western blot analysis. The expression level of TRPM4 in AAV2/9_CMV-TRPM4 injected mice varies from similar, slightly higher, to more than doubled compared to WT. We subdivided the animals in two qualitative groups: AAV2/9_CMV-TRPM4 injected WT mice with a comparable expression level compared to uninjected WT mice, and those with a clearly higher TRPM4 expression level then uninjected WT mice (see Table 2, based on the ratio TRPM4/Na,K-ATP-ase as Figure 3B). Table 2 illustrates that the majority of arrhythmic events after overexpression of TRPM4 are single VEBs. Also, the correlation between expression level and the number of arrhythmias is limited. Clearly, mice with the strongest arrhythmias are found in the group with moderate to strong overexpression of TRPM4. Episodes of non-sustained and sustained VT are only found in one mouse, which has strong overexpression of TRPM4. Likewise, one mouse with moderate overexpression of TRPM4 showed an exceptionally large number of conduction disturbances.

**TABLE 2:** Protein expression level and all arrhythmic events displayed by WT-mice subjected to TRPM4 expression during exercise-induced stress conditions.

		**Total # of arrhythmias**	**Ventricular arrhythmias**					**Conduction disturbances**		
				
				
**Mouse**	**Expression**		**Single**	**Couplet**	**Triplet**	**Non-**	**Sustained**	**AVB**	**AVB+**	**Sino-**
	**level**		**VEB**	**VEB**	**VEBs**	**sustained**	**VT**		**escape**	**atrial**
						**VT**			**beat**	**block**
45	0.1335	19	19	0	0	0	0	0	0	0
47	0.17226	79	49	0	4	1	0	0	0	25
79	0.18121	61	52	2	3	4	0	0	0	0
200	0.19084	77	58	0	0	0	0	1	0	18
41	0.21684	38	37	0	0	1	0	0	0	0
199	0.30936	51	12	0	0	0	0	10	7	22
78	0.32097	19	18	1	0	0	0	0	0	0
195	0.34474	533	43	0	0	0	0	429	26	35
197	0.37391	60	59	0	0	0	0	0	0	1
82	0.40812	77	57	5	0	0	0	2	1	12
196	0.41187	184	158	2	5	10	2	3	1	3
77	0.49927	16	16	0	0	0	0	0	0	0

*Mice are qualitatively subdivided in two groups, based on expression level of TRPM4 (TRPM4:Na,K-ATPase ratio, see also Figure 3B).*

Notably, injected mice with comparable TRPM4 expression as uninjected mice show a comparable number of VEB’s as injected mice with strong overexpression. This suggests that also the cell type and the distribution of cells that overexpress TRPM4 is an important determinant of sensitivity to arrhythmias. These factors cannot be controlled in the current experimental set-up.

## Discussion

In this study, we provide evidence that overexpression of TRPM4 is sufficient to increase the susceptibility of a living mouse to cardiac arrhythmias. We employed AAV2/9 viral vector transduction to deliver TRPM4 into mouse heart. Overexpression of TRPM4 does not affect the normal function (ECG parameters) of the heart in freely moving mice. Arrhythmic events are only significantly observed when provoked by an exercise stress test, and not in freely moving unprovoked mice.

Our results show a significantly increased number of VEBs following combined exercise- and ß-adrenergic stress in WT mice overexpressing TRPM4. In our experiments, the mean number of VEBs was increased to ∼50-fold compared to baseline following the exercise protocol, indicating that VEBs are specifically caused by the combination of an exercise stress and ß-adrenergic stimulation. Although single VEBs are considered to be a mild and non-lethal arrhythmic event in humans, it is obviously the predominant exertion of arrhythmic activity in mouse heart. As shown in previous studies, mice are relatively resistant to the development of ventricular tachycardia and ventricular fibrillation (for reviews see London, 2004; Choy et al., 2016). Variation in the susceptibility for complex arrhythmias clearly also depends on the mouse strain (Jelinek et al., 2018). In our study, mice are backcrossed to the C57Bl6/N background, in which complex arrhythmias have been difficult to elicit (Jelinek et al., 2018). Nevertheless, one mouse, injected with AAV2/9_CMV-TRPM4, developed ventricular fibrillation and idioventricular rhythm in the stress-test.

Combined exercise- and ß-adrenergic stress will promote mainly Ca^2+^ overload dependent triggered activity (Lehnart et al., 2008). This type of arrhythmias depends on abnormal intracellular Ca^2+^ release events in the cardiomyocyte. Considering that TRPM4 is a CAN cation channel, TRPM4 would contribute to abnormal depolarisation of cardiomyocytes in this condition, which will increase their excitability and their propensity to generate spontaneous APs. Furthermore, an increase in TRPM4 channel activity would increase Ca^2+^ dependent Na^+^ influx into cardiomyocytes. It is generally accepted that Na^+^ and Ca^2+^ handling is a major predisposing factor for life-threatening tachyarrhythmias (Wagner et al., 2015). Enhanced Na^+^ influx due to a gain-of-function of TRPM4 activity would lead to reduced Ca^2+^ extrusion by NCX function, secondary Ca^2+^ overloading in cardiomyocytes and triggered activity (Wagner et al., 2015).

A mathematical simulation suggested that heterogenous overexpression of TRPM4 will lead to conduction abnormalities, including all degrees of heart block (Type 1, 2, 3) (Gaur et al., 2018). This model suggests that TRPM4 is part of the background Na^+^ current (INa,b), that contributes to setting of the resting membrane potential and excitability of Purkinje cells (Gaur et al., 2018), though especially regional differences in overexpression would promote conduction disturbances. Though we expect that our experimental approach indeed results in heterogenous overexpression of TRPM4, and thus would represent an *in vivo* model of the mathematical assumptions in (Gaur et al., 2018), we did not observe an increase in conduction abnormalities in mice overexpressing TRPM4. Thus, it’s tempting to speculate that additional factors then gain-of-function of TRPM4 channel activity *per se* lead to cardiac conduction disorders. These might include cell specific effects or the interaction between TRPM4 and interacting partners. Currently none of these factors is well-defined.

Previously, AAV9 was used to deliver PKP2 R735X, a dominant negative mutation in the Plakophilin-2, into WT mice (Cruz et al., 2015). In these experiments, exercise triggered arrhythmogenic right ventricular cardiomyopathy (ARVC) in mice receiving the mutated form of PKP2. In another study, the Calsequestrin 2 (CASQ2) gene was delivered in CASQ2-knockout and CASQ2^*R33Q/*+^ mice mediated by AAV9 as a delivery mean to rescue their phenotype of Catecholaminergic Polymorphic Ventricular Tachycardia, an aggressive form of arrhythmia during exercise (Denegri et al., 2012, 2014). We delivered TRPM4 by AAV9 particles administered via tail vein injection. In line with previous observations, a large portion of viral particles accumulated in the liver (Inagaki et al., 2006; Zincarelli et al., 2008; Yang et al., 2009; Cruz et al., 2015). Endogenous TRPM4 expression level is low in mouse and human liver (Fonfria et al., 2006; Jang et al., 2012) and currently no clear indications point to a functional role of TRPM4 in the liver. For increased cardiac specificity of gene delivery, however, it seems mandatory to consider the use of a cardiac specific promoter, such as the α-MHC promotor region including its purine-rich negative regulatory (PNR) element (−344 to +119), as demonstrated by Aikawa et al. (2002), especially when opting for tail vein injection.

Mutations in the human *Trpm4* gene have been associated with hereditable conduction disorders (for reviews, see Abriel et al., 2012; Kruse and Pongs, 2014; Guinamard et al., 2015; Vennekens, 2018). First, a linkage study in a large South African pedigree with an autosomal-dominant form of progressive familial heart block type 1 (PFHBI) revealed a susceptibility locus on 19q13.33, a region which contains the TRPM4 gene (Kruse et al., 2009). Three additional mutations were linked to Isolated Cardiac Conduction Disorder (ICCD) in a French-Lebanese pedigree (Liu et al., 2010, 2013). Subsequently, Stallmeyer et al. (2012) tested a cohort of 160 unrelated patients with various types of inherited cardiac arrhythmic syndromes and detected six new mutations in patients with AV blocks and RBBB. Another cohort study screened 248 Brugada patients and revealed 11 additional mutations in 20 in patients not carrying mutations in the known susceptibility genes for Brugada syndrome (Liu et al., 2010). Daumy et al. (2016) identified two new mutations by applying targeted next-generation sequencing in 95 unrelated patients with PCCD. Finally, Hof et al. (2017) found four patients with TRPM4 mutations in a cohort of 178 LQTS patients, with no mutations in the three major LQTS genes (KCNQ1, KCNH2, and SCN5A). Whether any of the mutations in the TRPM4 gene are disease-causing, as well as their overall contribution to prevalence of cardiac arrhythmogenic disease is still unclear.

Taken together, our data provide the first evidence in a living animal that increased expression of TRPM4 is sufficient to increase the risk of stress-induced arrhythmias. Further research will need to clarify the precise mechanism, and establish whether blockade of TRPM4 prevents or suppresses arrhythmias.

## Data Availability

All datasets generated for this study are included in the manuscript and/or the supplementary files.

## Ethics Statement

All animal procedures were approved by the Ethics Committee of the KU Leuven.

## Author Contributions

AP, NS, and FV performed the experiments and analyzed the data. CVdH and RG assisted with the viral vector design, production, and transduction. SK and SP provided the technical support concerning mice and analysis of data, respectively. GVV performed and analyzed the *in vivo* luminescence imaging. AP and RV analyzed the data and wrote the manuscript. RV acquired funding and designed the experiments.

## Conflict of Interest Statement

The authors declare that the research was conducted in the absence of any commercial or financial relationships that could be construed as a potential conflict of interest.
